# Cost-effectiveness of linking HIV and family planning services to prevent unintended pregnancies among women living with HIV

**DOI:** 10.1371/journal.pone.0314308

**Published:** 2024-12-05

**Authors:** Beena Nitin Joshi, Himanshu Chaurasia, Revathy R., Siddesh Shetty, Namrata Kharat, Shahina Begum, Aniruddha Deshpande, Digambar Kangule, Shrikala Acharya, Parmod Deoraj, Pravin Sanap, Iranna Mashal

**Affiliations:** 1 HTA Regional Resource Hub, Department of Operational and Implementation Research, ICMR- National Institute for Research in Reproductive and Child Health, Mumbai, Maharashtra, India; 2 Department of Biostatistics, ICMR- National Institute for Research in Reproductive and Child Health, Mumbai, Maharashtra, India; 3 Maharashtra State Family Welfare Board Pune, Government of Maharashtra, Pune, Maharashtra, India; 4 Mumbai District AIDS Control Society, Mumbai, Maharashtra, India; 5 Maharashtra State AIDS Control Society (MSACS), Mumbai, Maharashtra, India; 6 Department of Operational and Implementation Research, ICMR- National Institute for Research in Reproductive and Child Health- HTA Regional Resource Hub, Mumbai, Maharashtra, India; Christian Medical College, INDIA

## Abstract

The incidence of unintended pregnancies and transmission of infection from mother-to-child could be averted by implementation of linked HIV care and family planning services (prong-2). The objective of the study was to assess the cost-effectiveness of strengthening prong-2 interventions through linked HIV-family planning services, to prevent unintended pregnancies among women living with HIV. A Markov decision analytical model was performed from a disaggregated societal perspective. A hypothetical cohort of married, sexually active women living with HIV in reproductive age, availing services from public health settings in India, were followed to estimate the costs and health outcomes. The intervention was strengthening linkage of HIV with family planning services through training healthcare providers and improving focus of dual methods of contraception. The standard of care with focus on condom promotion primarily for infection control was the comparator. The outcome was measured as Incremental Cost-effectiveness Ratio in terms of unintended pregnancies, live-births, abortions, maternal deaths and infant infections averted. We conducted Probabilistic Sensitivity Analysis to evaluate uncertainties. The intervention was found to be cost-effective. Among a cohort of 782107, 72604 unintended pregnancies, 41610 induced abortions, 17425 unintended live-births, and 8722 deaths were averted by the intervention. At 2% mother-to-child transmission rate, 2752 infant infections were averted. An incremental cost of INR 100000 (USD1272.3) could avert one death, two unintended live-births, five abortions and nine unintended pregnancies. Linking HIV and family planning services to prevent unintended pregnancy by use of dual contraception among women living with HIV is cost-effective.

## Introduction

According to the World Health Organization (WHO), 38.4 million people were living with HIV by the end of 2021 [1]. Around 1.3 million pregnant women were living with HIV the same year, increasing the risk of parent-to-child transmission [2]. WHO has recommended a four-pronged strategy to prevent and manage HIV. The first prong deals with the prevention of the infection; the second focuses on the prevention of unintended pregnancies through linking HIV care with family planning (FP) services, promotion of modern and dual contraception methods and training of healthcare providers, prong-3 is providing specific interventions to inhibit the transmission of the disease from the pregnant mother to her infant, and prong-4 is care, support, and treatment for infected mothers and children [3]. The Sustainable Development Goals (SDG) targets to end the AIDS epidemic [4] by 2030. To achieve this, strengthening all the prongs, along with addressing the high unmet need for FP [5] is vital.

According to the National AIDS Control Organization (NACO) of India, in 2021, 24.01 [19.92–29.07] lakh people are living with HIV (PLHIV) [6]. The national HIV incidence per 1000 uninfected population has plateaued in the country and has ranged from 0.06 (0.04–0.11) in 2015 to 0.05 (0.03–0.08) in 2021 [6]. According to the NACO Annual Technical Report of India HIV estimates 2017, 22677 [10927–40605] women living with HIV (WLHIV) and had given birth [7]. The lack of proper implementation of prevention of parent-to-child transmission (PPTCT) in India accounts for 8.76% (5.76–12.31) of HIV cases in the country [7]. It is higher in certain regions within the country like the eastern belt [10.83% (5.9–17.81)], Western region [7.6% (4.93–11.09)], and Maharashtra [3.57% (0.74, 10.08)] [7]. But similar to the total prevalence, the PPTCT need has also plateaued from 2018 [21517 (16888–27178)] to 2021 [20612 (16379–26359)], and no further reduction in these indicators had been achieved [6].

Since 2002, the PPTCT strategy was implemented in the country through which all registered pregnant women were freely tested for HIV from the antenatal clinics. Medications are provided free of cost to the beneficiary, and the infant receives anti-Retroviral (ARV) prophylaxis. WHO reported that with proper implementation of PPTCT strategies, the transmission rate can be brought down to 2% [8], from the current 5% [9] reported by NACO.

Various guidelines exist to address the sexual and reproductive health needs of PLHIV. But, indicators to measure it, like availability of contraception and the fulfilment of fertility desire is missing within these guidelines. Despite studies from India demonstrating a high prevalence of unmet need for family planning (FP) among WLHIV [10, 11], the programs tend to focus more on identifying and treating pregnant WLHIV and infants (prong 3 and 4) rather than emphasizing on prong 2 strategies like integrating HIV care and FP services [21]. Improved focus on prong 2 could reduce infection transmission, and prevent unintended pregnancies and HIV-positive birth [12–15]. This could also reduce the stigma related to the condition and improve the quality of life of WLHIV [16] and quality of care provided from the health facilities [17].

Various African studies have demonstrated the clinical and cost-effectiveness of this intervention [18, 19]. Even though evidence of its clinical-effectiveness exists in the Indian setting [20] there is no study on its cost-effectiveness. Every WLHIV has the right to accomplish her fertility desires like any other non HIV infected woman. Therefore, the policies and programs should be able to emphasize and address this unmet need to ensure availability of integrated HIV care and FP services to this vulnerable population. Even though the clinical effectiveness of the prong 2 strategies have been documented well, it is necessary to explore its cost-effectiveness, so that implementation of these interventions can be adapted into the existing programs [21].

India is a signatory of the 90:90:90 strategy by the United Nations [22]. The National Strategic Plan aims to make India AIDS-free with zero new AIDS infections, AIDS-related deaths, and AIDS-related stigma [22]. In this context, a health technology assessment on the cost-effectiveness of the prong-2 strategy by linkage of HIV care with FP services could help strengthen advocacy on the need to mainstream such interventions in the public health system from an equity perspective, and reduce the unmet need for contraception among the vulnerable PLHIV community. Therefore, this study aimed to assess the cost-effectiveness of strengthening prong-2 interventions through the provision of linked HIV-FP services to prevent unintended pregnancies among WLHIV. The research question for this study was proposed by the State Family Welfare Bureau Govt of Maharashtra.

## Methods

We developed a Markov decision analytical model using the principles of India HTAIn reference case [23]. We chose a disaggregated societal perspective for analysis. The population included a hypothetical cohort of 782107 [24], married, sexually active WLHIV in the reproductive age group (15–49 years) irrespective of the concordant/discordant spouse status, who were availing services from the Anti-retroviral Treatment (ART) Centre or Department of Obstetrics and Gynaecology (OBGYN) facilities in public health care settings, in India. We excluded women with stage IV HIV as per WHO criteria, and those who had attained menopause. The intervention was strengthening the linkage of HIV care with FP services through training of healthcare providers, assessment of unmet needs of FP, counseling, and providing linked services at the health facility along with timely monitoring and evaluation. The interventions were identified from the intervention package used in an RCT [20], which was formulated after carefully considering the gaps in the standard of care. The package included:

1. Training: One-day training of existing health care providers both from HIV and Family planning service divisions on assessing unmet need for contraception, counselling and promoting dual methods, assessing the eligibility for use of FP methods and provision of referral linkages and maintaining records of current contraceptive use, referred cases, methods accepted, unintended pregnancies, abortions etc.
2. Information, Education and Communication (IEC) activities: Develop and display of print material on importance of use of dual FP methods at both HIV and Family planning care facilities.
3. Counseling: Assessing unmet needs and promoting dual methods during regular visits of HIV Positive couples to HIV care centers for collecting their free ART drug supply.
4. Referral linkage to FP services: Facilitate referral for dual contraception within the hospital, to family planning centers, assisted with a referral slip.

The training would focus on sensitising the service providers on the rights and need to provide FP methods to PLHIV with focus on dual methods. The introduction of the WHO guidelines [25] for contraception would facilitate dispelling the misconceptions among service providers on the eligibility of WLHIV to use FP methods [20]. The current standard of care (SOC) that focuses predominantly on condom promotion for infection control without emphasis on dual methods of contraception and referral for FP was the comparator [20]. The effectiveness of the intervention was measured as incremental cost-effectiveness ratio (ICER) in terms of incremental cost per unintended pregnancies, unintended live-births, induced abortions, infant-HIV infections, and maternal deaths averted by implementing it. The cycle length was one year (nine months during pregnancy and three months postpartum) with a time horizon of 34 years.

### Model structure

**Fig 1:**
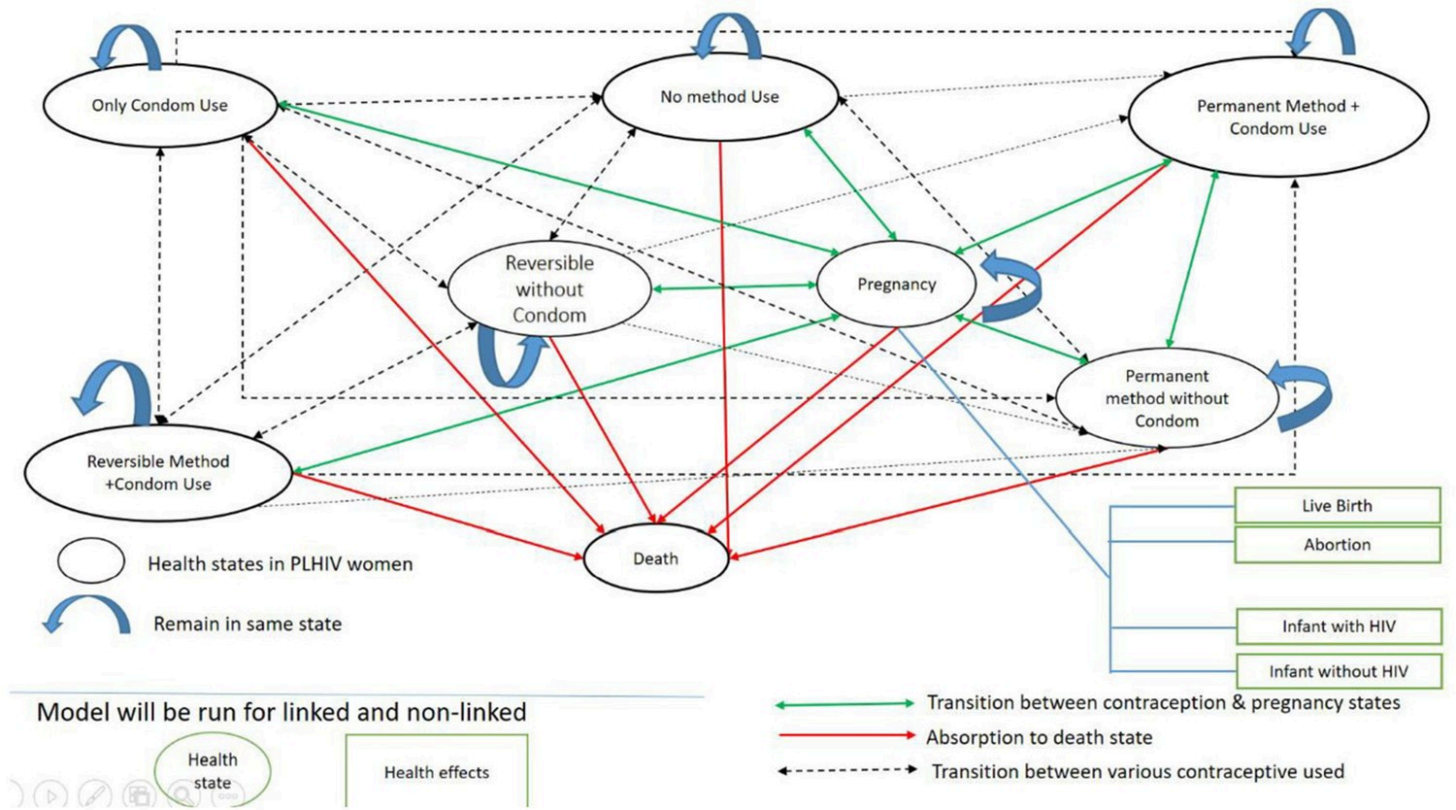
The Markov model. Markov model for women living with HIV in the reproductive age group using different methods of contraception and the possible outcomes.

The model was developed using Microsoft Excel 2016 and Microsoft Visual Basic for Application 7.1. The Markov model is presented as Fig 1. It began with a cohort of 782107 WLHIV that met the inclusion criteria. They transitioned through eight health states:

1. Not using any method of contraception
2. Using only condom
3. Using reversible contraception with condom (dual method-1),
4. Using reversible contraception without condom
5. Using irreversible contraception with condom (dual method-2)
6. Using irreversible contraception without condom
7. Pregnancy
8. Death

The cohort would move from one health state to another within their reproductive life span to progress into a pregnancy state with its possible outcomes and re-enter the model in a non-pregnant eligible state. Death was the absorptive state and was due to all-cause mortality along with HIV-specific mortality. Pregnancy could be intended/unintended and could result in live births (vaginal/C-section) or abortions. After birth, the infant would receive Anti-Retro Viral prophylaxis and will be tested for the infection at six months as per the existing standard of care. Post-test, the newborn could be diagnosed to be infected with HIV or not. We assumed that all WLHIV in the cohort either used modern contraception or were non-users even if they used less-effective or traditional methods.

### Probabilities

We extracted the age-specific proportion of WLHIV in various health states from the data obtained from the Maharashtra State AIDS Control Society (MSACS) for 2019–20. National Family Health Survey (NFHS) data was used to derive the contraceptive discontinuation rate, method failure rate, and switching rates specific to Maharashtra [26]. We obtained the input parameters to populate the model from primary cross-sectional studies, systematic reviews, scoping reviews and the Health Management Information System (HMIS) of the states’ AIDS Control Society (Tables 1 and 2).

**Table 1:** The probabilities used to populate the Markov model.

Input parameters	Health states	Value	Source
Proportion of WLHIV in various health states at the start of the model	Non user	54%	Maharashtra State AIDS Control Society- PLHIV data for 2019–20
	Condom user	29%	
	Reversible with condom user	11%	
	Reversible without condom user	0	
	Irreversible with condom user	0	
	Irreversible without condom user	0	
	Pregnant	4%	
	Death	3%	
Proportion of WLHIV on ART	With intended pregnancy	97%	Maharashtra State AIDS
	With unintended pregnancy	3%	Control Society- PLHIV data for 2019–20
Proportion of pregnant WLHIV	With intended pregnancy	49%	Dharak et al. [28]
	With unintended pregnancy	51%	
Proportion of WLHIV with live births	With intended pregnancy	60%	
	With unintended pregnancy	24%	
Proportion of WLHIV with still births	With intended pregnancy	10%	
	With unintended pregnancy	19%	
Proportion of WLHIV with abortions	With intended pregnancy	30%	
	With unintended pregnancy	57%	
**Input parameters**		**Value**	**Source**
Proportion of infants initiated on ARV prophylaxis		87.3%	Dharak et al. [28]
Proportion of infants tested for HIV within 6 months of birth		84.1%	
Proportion of infants diagnosed with mother on ART		4.35%	
Proportion of infants diagnosed with mother not on ART		0	Assuming that infants of mothers not on ART would not be receiving ART
Proportion of infants positive due to not being on ART		20%	National Technical Guidelines on Anti-Retroviral Treatment- 2018 [29]
Discount rate for outcome		3%	HTAIn Manual [23]
Discount rate for costs		3%	
Increment in dual method users in the intervention arm		22%	Joshi et al. [20]
Risk of transmission from Pregnant mother on ART to infants		2%, 5%	WHO [8], NACO [9] documents

**Table 2:** The cost parameters (health system cost and out-of-pocket-expenditure) used to populate the Markov model.

	Health system cost + Out of Pocket Expenditure (Median (IQR) in INR)	Health system cost + Out of Pocket Expenditure (Median (IQR) in USD)	Program cost (for IEC, training and incentive per beneficiary) INR (USD)	Intervention cost for linking HIV care with FP services INR (USD)
**Family planning methods used by the WLHIV**				INR 5000 (65.9 USD) per site [20]
Non-user	1200 (960, 1440)	15.27 (12.21, 18.32)	0	
Condom only user	1932.21 (1538.57, 2307.86)	24.582 (19.57, 29.362)	5.27 (0.1)	
Reversible contraception with condom user	2410.40 (1928.32, 2892.48)	30.66 (24.53, 36.8)	107.1 (1.4)	
Reversible contraception without condom	2168.05 (1734.44, 2601.66)	27.58 (22.07, 33.10)	104.8 (1.38)	
Irreversible contraception with condom	11454.60 (9163.68, 13745.52)	145.73 (116.59, 174.88)	2243.9 (29.6)	
Irreversible contraception without condom	11209.90 (8967.92, 13451.88)	142.62 (114.09, 171.14)	2239.2 (29.5)	
**Pregnancy outcomes**				
Pregnancy	10917.16 (8733.73, 13004.60)	138.89 (111.12, 164.45)	0.57 (0.008)	
Abortions	11928.27 (9542.61, 14313.92)	151.76 (121.41, 182.11)		
**Infant prophylaxis and testing**				
**Cost Parameters**	**INR**	**USD**	**Source**	
Cost for Infant prophylaxis	2450 (1960, 2940)	31.17 (24.94, 37.40)	Samudyatha et al. [30]	
Cost for Infant testing	30 (24, 36)	0.38 (0.31. 0.16)		

The proportion of non-users and only condom users after implementation of the intervention decreased by 10%, based on expert opinion. We obtained evidence from literature [20] that when the intervention is implemented, a 22% of increment would be visible in the health states with dual method use.

### Costs

We obtained the costs from primary cross-sectional studies and literature review. We conducted a mixed micro-costing study [27] to estimate the health system costs (HSC) for providing linked HIV and FP services to WLHIV, for the financial year 2019–20.

We also conducted a primary cross-sectional study to determine the out-of-pocket-expenditure (OOPE) of the WLHIV for availing HIV care and FP or pregnancy-related services. One hundred and ninety five WLHIV were recruited for this purpose, from three public health facilities of Mumbai, during April 2021 to February 2022. A detailed semi-structured questionnaire that collected information on the patient’s income and expenditure for availing these services was used. The questionnaire was administered in English and native language by trained research personnel. A written informed consent was obtained from the study participants. These primary studies were approved by the Institutional Ethics Committee. Additional ethical approvals and written permissions were taken from the study sites as well. The OOPE and HSC obtained from the primary studies are presented in Table 2.

The OOPE was measured as direct and indirect costs. The direct OOPE cost was highest among WLHIV who underwent abortion. Majority of the direct expense could be attributed to diagnostics and medications. Indirect cost was highest in WLHIV who had a C-section delivery. Majority of the indirect expense could be attributed to transportation and food. At 10% threshold value, 2% WLHIV incurred catastrophic expenditure for availing HIV care, FP and/ or pregnancy-related services.

The costs in the intervention and SOC arm are not very different from each other. This is because we are not proposing any major change in the functioning of existing health system. On the other hand, we are proposing to link these services by sensitizing and providing the additional FP training, identifying unmet need for contraception, counselling on dual methods, display of IEC material for linking HIV care with FP/ pregnancy-related services [27]. The incremental costs associated with these interventions were minimal. The results of the HSC analysis has been published as a manuscript [27].

### Analysis

Total cost for implementation of the intervention was obtained by adding the HSC, OOPE, program cost and intervention cost. We calculated the discounted and undiscounted ICER value (societal perspective) in terms of unintended pregnancies, unintended live-births, induced abortions, infant infections, and maternal deaths averted. The number of health outcomes averted was estimated by calculating the difference of the outcomes in the intervention and SOC arm. The cost-effectiveness threshold value was INR 145,742 (USD 2099; 1 USD = 78.60 INR) [31].

We conducted Probabilistic sensitivity analysis (PSA) to evaluate uncertainties in the model parameters, and used beta distribution for percentages, probabilities, and proportions and gamma distribution for costs and resource utilisation. The upper and lower limits of the parameters were either used as estimated from primary data or were assumed at 20% variation on each side. The Monte Carlo method simulated the results 1000 times. The median ICER, ICER plane, PSA plane, and Cost-Effectiveness Acceptance Curve (CEAC) planes were used to present the results.

We have adhered to the Consolidated Health Economic Evaluation Reporting Standards (CHEERS) checklist [32] for preparing the manuscript. The HTAIn Technical Appraisal Committee (TAC), Department of Health Research, India reviewed and approved this study.

## Results

The distribution of married WLHIV in the reproductive age group using various contraceptives was obtained from the MSACS data 2019–20. While the non-users were highest in the youngest age group (15–19 years), the percentage of condom users remained relatively stable throughout the age groups. The proportion of women preferring to use the reversible or irreversible method of contraception increased with age.

In India, among the cohort of WLHIV, 72604 more unintended pregnancies were averted in the intervention arm when compared to the SOC. Similarly, 41610 induced unintended abortions, 17425 unintended live births, 8722 maternal deaths were also averted. Considering an ideal PPTCT rate of 2% [8], among the unintended live-births, 2752 infant infections were averted. Similarly, as per programmatic coverage of 5% [9] PPTCT, 6880 infant infections were averted (Table 3).

**Table 3:** Number of averted health outcomes and incremental cost-effectiveness values in terms of unintended pregnancies, unintended live-births, induced abortions, and maternal deaths averted.

Health Outcomes	Number of averted cases for the cohort	Number of averted cases per person	ICER (Un discounted) INR (USD)	ICER (Discounted) INR (USD)
Unintended pregnancies averted	72604	0.092	27868 (354.6)	11435 (145.5)
Unintended, Live births averted	17425	0.022	116117 (1477.3)	47647 (606.2)
Induced Abortions averted	41610	0.053	48626 (618.7)	19953 (253.9)
Infant infections averted (2%)*	2752	0.004	-	-
Infant infections averted (5%)*	6880	0.009	-	-
Maternal deaths averted	8722	0.011	231988 (2951.5)	95192 (1211.1)

* At the World Health Organization reported ideal parent-to-child transmission rate of 2% and National AIDS Control Program reported parent-to-child transmission rate of 5%

**Fig 2:**
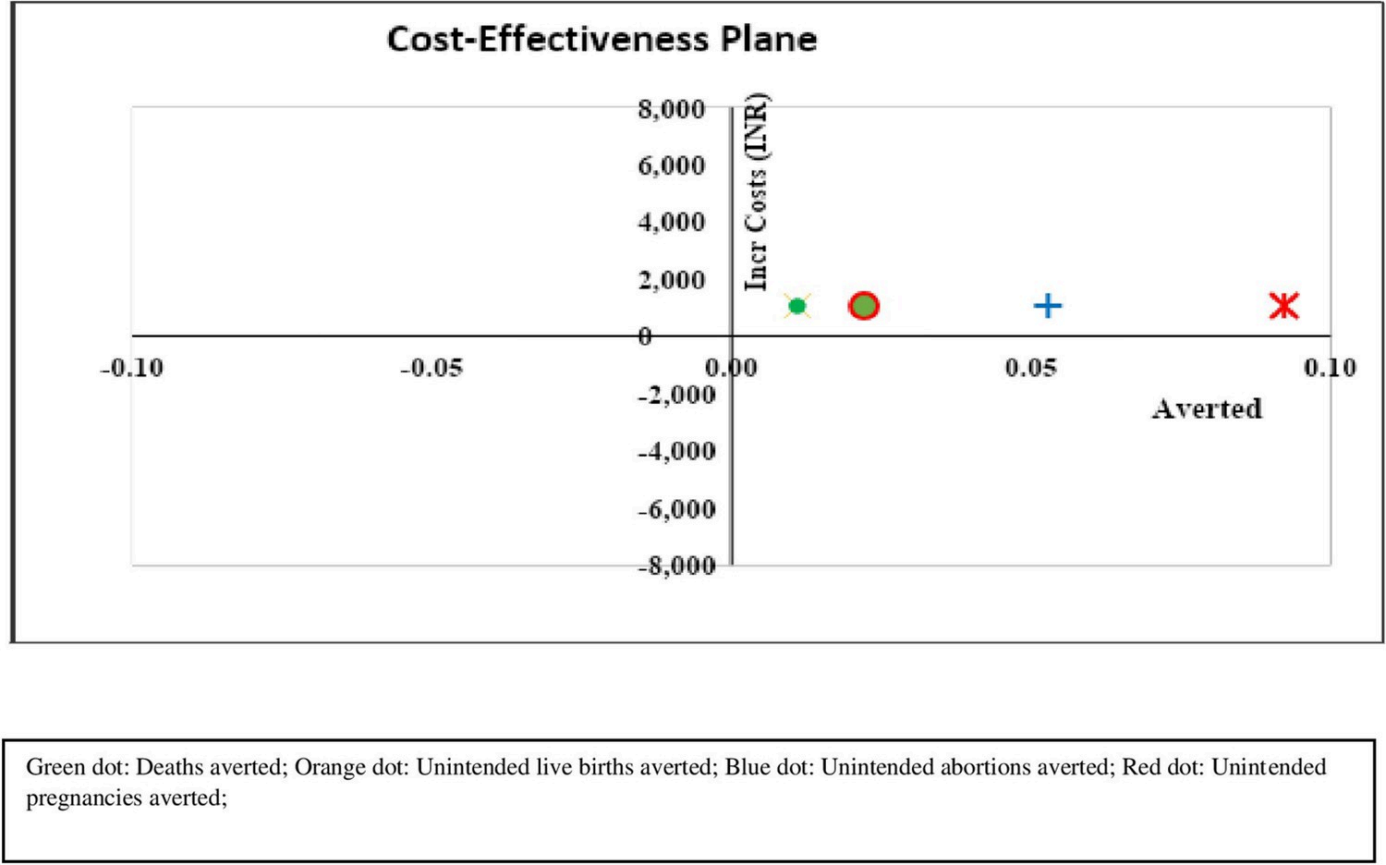
The ICER plane. The ICER plane depicts the maternal deaths averted, unintended pregnancies averted, unintended abortions averted, and unintended pregnancies averted. Green dot: Deaths averted; Orange dot: Unintended live births averted; Blue dot: Unintended abortions averted; Red dot: Unintended pregnancies averted.

The ICER was calculated in terms of unintended pregnancies, unintended live-births, induced abortions and maternal deaths averted. It was estimated that INR 11435 (USD 145.5) was required to avert one unintended pregnancy in the intervention arm. Similarly, INR 47647 (USD 606.2) was required to avert one unintended live-birth, INR 19953 (USD 253.9) to avert one induced abortion and INR 95192 (USD 1211.1) to avert one maternal death. All these incremental costs were less than the willingness to pay (WTP) threshold and therefore cost-effective. The ICER values are presented in Table 3. The ICER plane depicts that the intervention is cost-effective in terms of all the health outcome measures (Fig 2). At an incremental cost of INR 100000 (USD 1272.3), one death, two unintended live-births, five induced abortions and nine unintended pregnancies could be averted.

Regarding the PSA for each health outcome, 100% of the Monte Carlo simulations remained cost-effective in case of pregnancies averted; 95.7% remained cost-effective for induced unintended abortions averted; 71.1% remained cost-effective for unintended live births averted; and 57.1% remained cost-effective for deaths averted. The curve plateaus at about 90–96%, which is the maximum proportion of simulations that can be cost-effective in the model for these health outcomes.

## Discussion

WHO promotes a comprehensive approach to PPTCT. This strategy has four prongs that encompass a broad range of prevention, care, treatment, and interventions for support [3]. The present study is a health technology assessment that aims to estimate the cost-effectiveness of strengthening prong-2 interventions in public health settings in India. The intervention (linking HIV care with FP services with emphasis on dual method use) was compared with the SOC.

The modelling exercise found this intervention was cost-effective in terms of unintended pregnancies, induced abortions, unintended live-births, maternal deaths, and new infant infections averted. The costs associated with the intervention were mainly for the additional information, education, and counseling, and training activities and were not hugely different from the SOC. These findings appeared to be robust in the uncertainty analysis. This study demonstrated that integrating HIV and FP services is cost-effective in reducing new HIV infections among infants by averting unintended pregnancies.

Our study findings resonate with the results of previous research from Africa [18, 19, 33]. There were several methodological challenges highlighted in these previous studies and we have tried to address this in our methodology and analysis, as mentioned below.

A study from Kenya [18] in 2013 measured the cost, efficiency, and effectiveness of integrating FP and HI services. The study has concluded that it is inexpensive to integrate these services. This study also found the intervention to be feasible and efficient. Additionally, they reported that this intervention was a cost-efficient way to deliver contraceptive services to people living with HIV and could be cost-saving with a higher patient load. This study had raised concerns that it didn’t consider pregnancies averted while measuring the cost-effectiveness. We have tried to measure the same along with averted unintended live-births, induced abortions, HIV-positive infant births, and maternal deaths.

Another study [19] in 2006 assessed the cost-effectiveness of the intervention in terms of HIV-positive births averted, in a sub-Saharan population. They found that 28.6% more HIV-positive births were averted through FP programs in comparison to the nevirapine-oriented PPTCT program. This study raised concern about whether the proportions used for unintended pregnancies and unmet needs were conservative and an underestimation of the reality. We tried to address this by adhering to data from Indian settings for deriving the proportions and probabilities used in the model.

A Zambian study [33] in 2020 assessed the effectiveness of integrating FP services with HIV testing centers, with special emphasis on long-acting reversible contraceptives (LARC). The intervention was cost-saving in terms of cost per unintended pregnancy and perinatal HIV infection averted. This did not consider OOPE and could have underestimated their cost-effectiveness. To address this, we conducted a primary study to collect the OOPE by the WLHIV from Indian public health facilities.

During data collection for our primary studies, we observed the number of dual-method users was low in the health setting. Various Indian studies have highlighted the inadequate emphasis given to the promotion of dual methods among WLHIV [20, 34].

Despite trying to adhere to a comprehensive and robust methodology, this modelling exercise has a few limitations. The intervention could have more benefits, other than the outcomes that we have measured; therefore, the cost-effectiveness could have been under-estimated. Given the sparse literature evidence on many of the parameters required for building the model, we have used the available data which mainly comes from the state of Maharashtra. This could have limited the generalizability of the model results to the entire country. However, Maharashtra, a high-prevalence state, the results are generalizable only to states/areas with similar prevalence and fertility rates. A 2012 study [28] was the only study from India that reported the proportion of intended and unintended birth outcomes among WLHIV. This further highlights that the FP and pregnancy among WLHIV is a highly unaddressed issue in program settings as well as research, even today. Contraceptive use is dependent on women/ couple behaviour within the context of societal acceptance and quality of care available through the health system and therefore the probabilities derived from the literature are subjective. We have restricted our model to childbirth and therefore did not calculate the future costs of managing a child living with HIV.

## Conclusion

According to the latest recommendations of WHO [35] viral suppression in pregnant WLHIV to undetectable levels could reduce perinatal transmission to <1%. These recommendations have been recently introduced and are yet to demonstrate significant impact on mother-to-child transmission rates. As discussed above, the programmatic data of India show 5% PPTCT rates in the country [9]. Nevertheless, preventing unintended pregnancies could reduce the burden on the health of the WLHIV along with the cost burden on the health system to manage pregnancy, post-partum care and diagnosis and management of the infant. Access to integrated delivery services could facilitate accomplishing the last difficult mile to reach the Sustainable Development Goal target of ending epidemics like AIDS by 2030 [4]. Access to effective contraception is a matter of right for WLHIV, as is for the general population. HIV programs must invest in effective measures to prevent discrimination against this vulnerable population. Even though a logical conclusion, many studies have failed to emphasize the aspect of averting unintended pregnancies within the AIDS control program. This study presents evidence on the cost-effectiveness in promoting various FP methods in general and dual methods in specific, among WLHIV.

The research question for the study being proposed by the Government indicates that this is an identified programmatic gap with a felt need to integrate the services. “Cost-effectiveness” is not the only parameter that would determine the implementation of integrated care within the public health system. However, as in all cases of using evidence of cost effectiveness analysis to promote a health technology including a program, this remains as one of the tools in the advocacy basket apart from other factors like political will to address equity and universal health coverage in policy decision making.
